# Supplementary material for: Micropublications: a semantic model for claims, evidence, arguments and annotations in biomedical communications
Source: J Biomed Semantics. 2014 Jul 4;5:28. doi: 10.1186/2041-1480-5-28 (PMC4530550; doi:10.1186/2041-1480-5-28)
Supplement: Additional file 1 — OWL Vocabulary and RDF Examples. [file 2041-1480-5-28-S1.pdf]

# Class, Predicate and Rule Definitions for Micropublications

## Additional Material 1 for:
*Micropublications: a Semantic Model for Claims, Evidence, Arguments and Annotations in Biomedical Communications*

Tim Clark[1,2,3]*, Paolo N. Ciccarese[1,2], Carole A. Goble[3]

[1] Department of Neurology, Massachusetts General Hospital
55 Fruit Street, Boston MA 02114, USA

[2] Harvard Medical School
25 Shattuck Street, Boston, MA 02115 USA

[3] School of Computer Science, University of Manchester
Oxford Road, Manchester M13 9PL, UK

* Corresponding author

Email addresses:
TC: tim_clark@harvard.edu; clarkt@cs.manchester.ac.uk
PNC: paolo.ciccarese@gmail.com
CAG: carole.goble@cs.manchester.ac.uk

## A.1 Class, Predicate and Rule Definitions for Micropublications

Class definitions follow. We divide them for convenience into groups: Artifacts and Representations; Scientific Evidence; Argumentation; and Representative Statements. An asterisk (*) prior to a predicate indicates a datatype property; all other are object properties.

### *A.1.1 Class Definitions*

#### *A.1.1.1 Activities, Agents, Artifacts and Representations*

- *Agent* ::= an Entity that makes, modifies, consumes or uses an *Artifact*; *Artifacts* can be connected to Agents by the *attributedTo* property, and to an *Attribution* by the *attributionOfAgent* property.
  - subclass of: *Entity.*
- *ArticleText* ::= a URI identifying the text of a scientific article.
  - subclass of: *Representation.*
- *Artifact* ::= a produced or acquired material object, energy flow or set of processes;
  - Examples: reagents, instruments, video feeds, documents, ontologies, email, computers, language, music, dance, a football game, a no-foam skinny latte, a laboratory, an experiment, a University, an oil refinery, 120V-60Hz electrical current; the Internet; a kindergarten; or any cultural or technological thing or process created by humans;
  - subclass of: *Entity.*
  - Predicates:
    - *hasAttribution*: domain Artifact; range: Attribution.

        - *representedBy*: domain Artifact; range: Representation; inverse of *represents*.
        - *attributedTo*: domain Artifact; range: Agent.
            - subproperties*: authoredBy, *authoredOn, curatedBy, *curatedOn, editedBy, *editedOn, publishedBy, *publishedOn*.
- *Attribution* ::= a Representation showing how, by whom, and in what context, an Artifact was made, modified, and/or acquired;
    - subclass of: *Representation.*
    - Predicates:
        - *hasAttribution*; domain Artifact; range: Attribution; subproperty of *supports*.
        - *attributedAsAgent*: domain Attribution; range: Agent.
        - Subproperties*:*
            - *attributionAsAuthor, attributionAsCurator, attributionAsEditor, attributionAsPublisher.*
        - Property Chains:
            - *hasAttribution* and *attributionAsAuthor* => *authoredBy*;
            - *hasAttribution* and *attributionACurator* => *curatedBy*;
            - *hasAttribution* and *attributionAsAuthor* => *editedBy*;
            - *hasAttribution* and *attributionAsAuthor* => *publishedBy*.
        - **atTime*: domain Attribution; range: dateTime
- *Entity* ::= any Thing, real or conceptual, which may enter into a discussion.
    - Predicates:
        - *representedBy:* domain Entity; range: Representation.
- *Qualifier* ::= a Sentence modifying or particularizing the epistemic strength, scope, or other aspect of meaning, of a Statement.
    - subclass of: *Sentence.*
    - Subclasses: *Reference*, *SemanticQualifier*.
    - Examples: Hedging phrases are modifying Qualifiers in natural language, e.g.: "it seems to us", "we hypothesize". Folksonomic tags are Qualifiers.
    - Predicates:
        - *qualifies*: domain: Qualifier; range: Statement; inverse of *qualifiedBy*.
- *Referenc*e ::= a Qualifier purporting to indicate a certain document; when deployed as a Qualifier of a Statement, indicating that support for the Statement may be found in the referenced document.
    - Example:
        - "Spilman et al. 2010 ";
        - "URI: http://dx.doi.org/10.1371%2Fjournal.pone.0009979";
        - "DOI: 10.1371/journal.pone.0009979";
    - Subclass of: *Qualifier*;
    - Predicates:
        - *supports*: domain: Reference; range: Statement; inverse of *supportedBy*.
- *Representation* ::= a finite, ordered non-null collection of digital Artifacts representing material objects, events, processes, states of affairs, and/or symbols. Furthermore, quoting Rees 2012 [1], *Representation* "is a term of art meaning an octet sequence (the "content") together with metadata, such as media type, that directs the interpretation of the content".

  - Examples: a sentence, an equation, an ontology, a document, a photograph, an audio or video recording, a bibliographic reference;
  - Subclass of: *Artifact*.
  - Predicates:
    - *supports*: domain: Representation; range: Representation; inverse of *supportedBy;* transitive property.
    - *challenges*: domain: Representation; range: Representation; inverse of *challengedBy;* superPropertyOf *directlyChallenges* and *indirectlyChallenges;*
    - *indirectlyChallenges*: domain: Representation; range: Representation; inverse of *indirectlyChallengedBy;* superPropertyOf property chain (directlyChallenges, supports);
    - *directlyChallenges*: domain: Representation; range: Representation; inverse of *directlyChallengedBy;*
    - *discusses:* domain: Representation; range: Representation; inverse of *discussedBy*.
    - *represents*: domain: Representation; range: Artifact; inverse of *representedBy*.
    - **value*: domain: Representation; range: none.
- *SemanticQualifier* ::= a Qualifier belonging to a formal vocabulary or ontology.
  - Subclass of: *Qualifier*.
- *Sentence* ::= meaningful symbolic Representation, well-formed in its domain; may consist of a single word or phrase; does not need to be a "complete sentence" with subject, predicate, object, etc.;
  - Examples: "This is a sentence"; "a sentence"; "sentence"; "$R \subseteq \Phi \times \Phi$"; "CHEBI: 9168"; "Spilman et al. 2010" : "£3.65"; "¬";
  - Subclass of: *Representation*.
- *Statement* ::= a declarative Sentence;
  - Examples: "This is a statement."; "It's time for lunch."; "$R \subseteq \Phi \times \Phi$";
  - Subclass of: *Sentence*
  - Predicates:
    - *arguedBy*: domain: Statement; range: Micropublication; if a Statement is *arguedBy* a Micropublication, it is implicitly a Claim. There will be one and only one Claim in any Micropublication.
    - *qualifiedBy*: domain: Statement; range: Qualifier; inverse of *qualifies*
    - **statement:* domain: Statement; range: string.

#### *A.1.1.2 Scientific Evidence: Data, Method, Procedure and Material*

  - *Data ::=* one or more *Representations* of the results of observation and/or experiment*;*
    - subclass of: *Representation.*
  - *Method ::=* a specification or description of a process for obtaining a set of *Data*, including instructions for reproducibly making, obtaining, and/or employing the necessary *Artifacts;*
    - subclass of: *Representation.*
  - *Material* ::= a *Representation* of an *Entity* or *Artifact*, specified in a *Method*, such as physical reagents, tools, instruments, subjects, software, etc., which may be used by *Agents* in creating *Data;*
    - subclass of: *Representation*

### *A.1.1.3 Argumentation*

- *Micropublication* ::= a formal argument structure in metadata, which may annotate one or more arguments which are themselves expressed either in text or a formal language; subclass of Representation. Micropublications may annotate, e.g., a full text scientific article, a PubMed entry, or another Micropublication. They may also stand on their own.
    - A Micropublication consists of
        - the Micropublication's *Attribution*, i.e. of this formalization of the argument's content;
        - the *Claim* ::= a principal Statement argued for by a Micropublication; the principal Statement of the Micropublication's formalized argument;
        - the Claim's *Attribution* (to the original author);
        - the *SupportGraph* ::= the set of all Representations supporting its Claim which are *elementsOf* a Micropublication;
        - the ChallengeGraph ::= the set of all Representations challenging its Claim which are *elementsOf* a Micropublication.
    - subclass of: *Representation.*
    - Predicates:
        - *argues*: domain: Micropublication; range: Statement; inverse of *arguedBy;*
        - *asserts:* domain: Micropublication; range: Representation; inverse of *assertedBy*; subclass of *hasElement;*
        - *hasElement*: domain: Micropublication; range: Representation; inverse of *elementFor*; subproperties: *asserts*, *quotes*;
        - *hasAttribution:* domain: Micropublication; range: Attribution; subPropertyOf *supportedBy*.
        - *quotes:* domain: Micropublication; range: Statement; inverse of *quotedBy;* subclass of *hasElement*.
    - Support and Challenge Graphs
        - Support and Challenge Graphs are defined by query on the relations *hasSupportGraphElement* and *hasChallengeGraphElement.*
        - *hasSupportGraphElement* relates the Micropublication to all Representations which are an *elementOf* the Micropublication, and have the relation *supports* to the Micropublication's Claim.
        - *hasSupportGraphElement* relates the Micropublication to all Representations which are an *elementOf* the Micropublication, and have the *challenges* relation to the Micropublication's Claim.

### *A.1.1.4 Representative or Canonical Statements (Holotypes)*

Because there are many similar Statements in the scientific literature expressing the same, or nearly the same, meaning, we need a way to group them into classes of "similar-enough" meaning. We call these similar-enough statements *similogs*, by analogy with sequence homologs in molecular biology. A set of similogs constitutes a *Similarity Group*.

Such groups may be represented by a selected single statement which stands for the group. This is a method of convenience which avoids combinatorial explosion when defining statement relationships. Biologists do this in defining species: they "collect" a *Holotype*, or representative "specimen" of a group, which is then described in lieu of the group.

Determining what constitutes enough similarity to declare Statements similogs, depends entirely upon the domain and application, and may require human judgment. It is an assignment of "close enough" similarity in meaning.

- *Holotype* ::= a Statement which expresses the same or substantially similar meaning to one or more other Statements, and which is selected for convenience to represent them.
    - Predicates:
        - *hasHolotype:* domain and range: Statement; inverse *holotypeFor*.
    - Example:
      The following two Statements are highly similar in their meaning, one being derived from the other via paraphrase, and either may be selected as *holotypeFor* the other:
        - de Calignon et al. 2012, Neuron [2]: *"brain extracts from neurofibrillary tangle-bearing mouse brain injected in wild-type tau-expressing mice induces seeding of tau fibrils in neurons*" (citing Clavaguera et al. 2009);
        - Clavaguera et al. 2009, Nature Cell Biology [3]: *"injection of brain extract from mutant P301S tau-expressing mice into the brain of transgenic wild-type tau-expressing animals induces assembly of wild-type human tau into filaments"*
    - Discussion:
      Given two similar Statements $s_i$ and $s_j$, the degree of similarity of meaning between them may be defined algorithmically, for example by algorithmic clustering or linguistic analysis; by citation inheritance (see (a) below); by observation and human judgement; or by any other useful method.

      The set of similar Statements for si∈S, given a binary relation $\sim(s_{i,} s_j)$, is the *equivalence class* $[s_i] = \{ s_i \in S \mid \sim(s_{i,} s_j) \}$.

### *A.1.2 Predicate Definitions*

- *attributionOfAgent*: domain: Attribution; range: Agent.
- *argues*: domain: Micropublication, range: Statement; inverse of *arguedBy,* subproperty of *supportedBy*.
    - When a Statement is the principal assertion of a Micropublication, the Micropublication *argues* the Statement, and the Statement is termed the *Claim* for that Micropublication.
    - *arguedBy*: inverse of argues; subproperty of *supports*.
- *asserts:* domain: Micropublication; range: Representation; inverse of *assertedBy*.
    - A Micropublication *asserts* Representations for which its publishing authority takes direct responsibility.
    - *assertedBy*: inverse of *asserts;* concept from [4].
- *attributionAsAuthor*: domain: Representation; range: Attribution; subproperty of *hasAttribution*.
- *attributionAsCurator*: domain: Representation; range: Attribution; subproperty of *hasAttribution*.
- *attributionAsEditor*: domain: Representation; range: Attribution; subproperty of *hasAttribution*.
- *attributionAsPublisher*: domain: Representation; range: Attribution; subproperty of *hasAttribution*.
- *attributionOfAgent*: domain: Representation; range: Attribution; subproperty of *hasAttribution*.

- *challenges*: domain & range: Representation; inverse of *challengedBy*;
    - A Statement *challenges* another Statement when it asserts or implies that the second Statement *challengedBy* the first Statement, is false. This can be by direct challenge (*directlyChallenges*) or indirect challenge (*indirectlyChallenges*).
    - *challengedBy*: inverse of *challenges*.
- *dataSupports*: domain: Data; Range: Method; inverse of *supportedByData*; subproperty of *evidenceSupports*.
- *directlyChallenges*: domain and range: Representation; inverse of *directlyChallengedBy*;
    - a Representation which *directlyChallenges* another, says that it is false.
    - *directlyChallengedBy*: domain and range: Representation; inverse of *directlyChallenges.*
- *discusses*: domain: Statement; range: Representation; inverse of *discussededBy;*
    - A Representation discusses another when it comments neutrally upon it.
    - *discussedBy*: domain: Representation; range: Statement; inverse of *discusses.*
- *evidenceSupports*: domain: Data, Method, Material; range: Representation; inverse of *supportedByEvidence*; subproperty of *supports*.
    - *subproperties*: *dataSupports*, *methodSupports*.
- *hasAttribution*: domain: Represenation; range: Attribution; subproperty of *supportedBy*; subproperties: *attributionAsAuthor, attributionAsCurator, attributionAsEditor, attributionAsPublisher*.
- *holotypeFor*: domain and range: Statement; inverse of *hasHolotype.*
    - The relation *holotypeFor* identifies a single Statement $s_i \in S$ taken as representative of the HomologyGroup S as a whole; subproperty of *represents;* inverse *hasHolotype*.
    - *hasHolotype:* domain and range: Statement; inverse of *holotypeFor.*
- *indirectlyChallenges*: domain & range: Representation; inverse of *indirectlyChallengedBy*.
    - a Representation which challenges any Representation in the chain of support for another Representation, *indirectlyChallenges* that Representation.
    - *indirectlyChallengedBy*: domain and range: Representation; inverse of *indirectlyChallenges*.
- *methodSupports*: domain: Method; range: Representation; inverse of *supportedByMethod;* subproperty of *evidenceSupports*.
- *produces*: domain: Method; range: Data; inverse of *producedBy*.
    - a Method *produces* Data when that Data is an output created by applying the procedures described by the Method.
- *qualifies*: domain: Sentence; range: Statement; inverse of *qualifiedBy*.
    - a Sentence qualifies a Statement when it modifies its range, scope, or epistemics.
    - Example: the Qualifier "it has been asserted that" *qualifies* (hedges) the Statement "Rapamycin inhibits the mTOR pathway".
    - *qualifiedBy*: domain: Statement; range: Sentence; inverse of *qualifies*.
- *quotes*: domain: Micropublication; range: Representation; inverse of *assertedBy*.
    - A Micropublication *quotes* Representations that it borrows, but for which its publishing authority takes indirect responsibility, e.g. those *asserted* in another Micropublication.
    - *quotedBy*: inverse of *quotes*; concept adapted from [4].

- *represents*: domain: Representation; range: Artifact; inverse of *representedBy*.
    - A Representation *represents* an Artifact when it communicates one or more properties of the Artifact to a suitable audience.
    - *representedBy*: inverse of *represents*.
- *supports*: domain and range: Representation; inverse of *supportedBy;* subproperties *arguedBy, evidenceSupports*.
    - A Representation *supports* another Representation, when the second Representation provides a warrant for belief in the first.
    - Subproperties: *argues*, *hasAttribution*.
    - Examples:
        - Methods may *supports* Data. The description of the Methods by which the Data were obtained supports belief in the Data.
        - A Statement *supports* another Statement by lending credence to it.
        - The Attribution of a Statement *supports* its truth or accuracy, by indicating who said it, who reviewed and/or published it, and in what context.
        - The Attribution of Methods *supports* them, by indicating where they originated, were obtained, or were previously used or validated.
    - *supportedBy*: domain and range: Representation; inverse of *supports*.
- *usedIn*: domain: Material; range: Method; inverse of *uses*;
    - A Material is *usedIn* a Method, when the Entity *representedBy* the Material is a required component in the process the Method describes.

### *A.1.3 Rule Definitions*

This vocabulary is associated with the following mandatory rules (see Section 5.1 for SWRL implementation details).

- **Support and Challenge Graph Rules**:

These two rules are essential for proper functioning of the model.

- An *elementOf* any Micropublication, which also *supports* its Claim, has the property *supportGraphElementOf* with the Micropublication as object.
- An *elementOf* any Micropublication, which also *challenges* its Claim, has the property *challengeGraphElementOf* with the Micropublication as object.

- **Attribution Timestamp Rules:**

These four rules are for convenience, so that dates of authorship, editing, etc. may be assigned directly to an Artifact.

If an Attribution is instantiated with an associated *atTime* property, that *atTime* value then also becomes the value of an associated dateTime property of the form *<action-past-tense>On*, asserted on the Entity being attributed, and corresponding to the *attributionAsAuthor (authoredBy)*, *attributionAsCurator (curatedBy)*, *attributionAsEditor (editedBy)*, and *attributionAsPublisher* (*publishedBy*) properties, as follows:

- *attributionAsAuthor* → *authoredOn*
- *attributionAsCurator* → *curatedOn*
- *attributionAsEditor* → *editedOn*
- *attributionAsPublisher* → *publishedOn*

## REFERENCES

1. Rees J: **Understanding URI Hosting Practice as Support for URI Documentation Discovery**. In., Editor's Draft 29 February 2012 edn: World Wide Web Consortium; 2012.
2. de Calignon A, Polydoro M, Suárez-Calvet M, William C, Adamowicz David H, Kopeikina Kathy J, Pitstick R, Sahara N, Ashe Karen H, Carlson George A *et al*: **Propagation of Tau Pathology in a Model of Early Alzheimer's Disease**. *Neuron* 2012, **73**(4):685-697.
3. Clavaguera F, Bolmont T, Crowther RA, Abramowski D, Frank S, Probst A, Fraser G, Stalder AK, Beibel M, Staufenbiel M *et al*: **Transmission and spreading of tauopathy in transgenic mouse brain**. *Nat Cell Biol* 2009, **11**(7):909-913.
4. Bizer C: **Semantic Web Publishing Vocabulary**. In.; 2004.
