# Supplementary material for: Micropublications: a semantic model for claims, evidence, arguments and annotations in biomedical communications
Source: J Biomed Semantics. 2014 Jul 4;5:28. doi: 10.1186/2041-1480-5-28 (PMC4530550; doi:10.1186/2041-1480-5-28)
Supplement: Additional file 2 — Class, Predicate and Rule Definitions for Micropublications. [file 2041-1480-5-28-S2.pdf]

OWL Vocabulary and RDF Examples

Additional Material 2 for:
Micropublications: a Semantic Model for Claims, Evidence, Arguments and Annotations in Biomedical Communications

Tim Clark[1,2,3]*, Paolo N. Ciccarese[1,2], Carole A. Goble[3]

[1] Department of Neurology, Massachusetts General Hospital
55 Fruit Street, Boston MA 02114, USA

[2] Harvard Medical School
25 Shattuck Street, Boston, MA 02115 USA

[3] School of Computer Science, University of Manchester
Oxford Road, Manchester M13 9PL, UK

* Corresponding author

Email addresses:
TC: tim_clark@harvard.edu; clarkt@cs.manchester.ac.uk
PNC: paolo.ciccarese@gmail.com
CAG: carole.goble@cs.manchester.ac.uk

A.2 OWL Vocabulary and RDF Examples

Micropublications may be represented using an OWL vocabulary, **mp**, available at http://purl.org/mp.

We clarify OWL-specific aspects of this vocabulary, below in Section 2.1.

Example RDF, in Turtle format, for Examples 1, 3, 5 (**Figures 7, 9, 13)** is shown below in sections 2.2 through 2.4. Section 2.5 shows details of how micropublications and nanopublications may be interfaced in a common model using the mp:supports and mp:argues relations (**Figure 20**).

*A.2.1 A Discussion of the **mp** OWL Vocabulary*

A few points about implementation of this model in OWL are worth pointing out here for clarity.

A.2.1.1 Entity, Agent, Attribution and their Properties

We reuse the classes *Entity*, *Agent* and *Attribution*; and the properties *wasAttributedTo (*via the subproperty *attributedTo)*, *hasAttribution*, and *agent* (via the subproperty *attributionOfAgent*) from the W3C Provenance Ontology (PROV-O) [1]. Every class in our model is a subclass of *prov:Entity*. Every attributed action in our model is performed by some *prov:Agent*.

We define subproperties for the PROV-O property *hasAttribution* for certain specific roles attributed to an Agent: *attributionAsAuthor, attributionasPublisher, attributionAsCurator, attributionAsEditor*. These can be added to as necessary.

We also define the superproperties *authoredBy, editedBy, publishedBy, curatedBy*, as superproperties of property chains on *attributionAsAuthor…etc.* and *attributionAsAgent.* These

superproperties (of property chains) are in the spirit of PAV, the Provenance, Authoring and Versioning Ontology [2]; two of them (*authoredBy* and *curatedBy*) reoccur in PAV.

PROV-O takes a more elaborate path to achieve role definitions, which we believe is unnecessary in the present context, although well-adapted for detailed workflows. If desired, however, our approach may readily be harmonized with the full machinery of PROV-O.

A.2.1.2 Representations as Elements of a Micropublication

The set of Representations in the model of a single Micropublication is jointly defined by the properties `asserts` and `quotes`, which have the superproperty `hasElement`; and so the set of Representations belonging to (`elementOf;` inverse `of hasElement`) a Micropublication correponds to Φ in the mathematical definition in section 3.2.3.

A Representation may be `assertedBy` only one Micropublication. It may be `quotedBy` many Representations; but not both `assertedBy` and `quotedBy` any, as these properties are disjoint.

A.2.1.3 The mp:value Property

The `mp:value` datatype property represents the literal content of a Representation. It has two subproperties: `mp:citation` and `mp:statement`. If a Representation has the `mp:statement` property with some string as its object, it is a Statement. If it has the `mp:citation` property it is a Reference.

A.2.1.4 Claims and Support / Challenge Graphs

We define the principal Claim as object of the `argues` predicate on a Micropublication. The Representations $\in \Phi$ for that Micropublication are defined by the predicate *hasElement*. Those which also have `supports` (and possibly `challenges`) relations to the Claim are made objects of the predicate `hasSupportGraphElement` (and possibly, `hasChallengeGraphElement)`, for that Micropublication, using DL-Safe rules implemented in the Semantic Web Rule Language (SWRL) [3, 4].

A.2.1.5 A Property Chain Underlies the `challenges` Property

Unlike `supports`, the `challenges` property is not transitive. But we want it to propagate across the network of relations. If I challenge the Method published by Bob, and used by Mary in some other publication to derive her Data, this challenge should propagate up to the central Claim of Mary's study, because we have undercut its support. We accomplish this by defining the properties `directlyChallenges`, and `indirectlyChallenges`. A `directlyChallenges` property is just what it implies: directly asserting that something is false.

That thing we directly challenged may *`supports`* (transitively or otherwise) some Statement or Claim or Data elsewhere, and so the `indirectlyChallenges` property will be entailed as a property chain on `directlyChallenges` and `supports`. Lastly, the `challenges` property is entailed as superproperty of `directlyChallenges` and `indirectlyChallenges`, also entailing instantiation of the `ChallengesGraph`.

A.2.1.6 DL Safe Rules

The (DL-Safe) rules associated with this model are given here using the Manchester vocabulary of SWRL.

- **Support and Challenge Graph rules**:
  - An *elementOf* any Micropublication, that also *supports* its Claim, has the property *supportGraphElementOf* connecting it to the Micropublication.

        - elementOf(?r, ?m), supports(?r, ?m) -> hasSupportGraphElement(?m, ?r)
    - An *elementOf* any Micropublication, that also *challenges* its Claim, has the property *challengeGraphElementOf* connecting it to the Micropublication.
        - challenges(?r, ?m), elementOf(?r, ?m) -> hasChallengeGraphElement(?m, ?r)
- **Attribution *atTime* rules:** If an Attribution is instantiated with an associated *atTime* property, that *atTime* value is the value of an associated datatype property:
    - ***authoredOn***: attributionOfAgent(?b, ?c), attributionAsAuthor(?a, ?b), atTime(?b, ?t) -> authoredOn(?a, ?t)
    - ***curatedOn***: attributionOfAgent(?b, ?c), attributionAsCurator(?a, ?b), atTime(?b, ?t) -> curatedOn(?a, ?t)
    - ***editedOn***: attributionOfAgent(?b, ?c), attributionAsEditor(?a, ?b), atTime(?b, ?t) -> editedOn(?a, ?t)
    - ***publishedOn***: attributionOfAgent(?b, ?c), attributionAsPublisher(?a, ?b), atTime(?b, ?t) -> publishedOn(?a, ?t)

Other aspects of the OWL ontology are relatively straightforward, correspond closely to the model, and are documented as annotation properties.

A.2.2 RDF for Example 1, Figure 7

```
@prefix     xsd:  <http://www.w3.org/2001/XMLSchema#> .
@prefix     rdf:  <http://www.w3.org/1999/02/22-rdf-syntax-ns#>.
@prefix     rdfs: <http://www.w3.org/2000/01/rdf-schema#> .
@prefix     rdfg: <http://www.w3.org/2004/03/trix/rdfg-1/>.
@prefix     dct:  <http://purl.org/dc/terms/> .
@prefix     foaf: <http://xmlns.com/foaf/spec/0.98#> .
@prefix     prov: <http://www.w3.org/ns/prov#> .
@prefix     obo: <http://purl.obolibrary.org/obo#> .
@prefix     mp:   <http://purl.org/mp/> .
@prefix     :     <http://www.example.com/micropublication/ex1/> .

{

### Agents
:ClarkT     a     mp:Agent, prov:Person, foaf:Person;
            foaf:name   "Tim Clark";
            foaf:mbox_sha1sum
      "d9c9efcbcb2877d085cff13ec8b748772fec0ed6" .

:SpilmanP   a     mp:Agent, prov:Person, foaf:Person;
            foaf:name   "Patricia Spilman";
            foaf:mbox_sha1sum
      "6b4cacd4bc4de8d5d45903c29dec30eba1d696d3" .

### Micropublication :MP1
:MP1= {:MP1 a mp:Micropublication ;
            rdfs:label        "MP1: MP Example 1";
            mp:asserts        :C1,:A_C1,:Ref5;
            mp:quotes         obo:CHEBI_9168, obo:INO_0000736;
            mp:argues         :C1;
            mp:attributionAsAuthor :A_MP1 ;
            mp:represents
      <http://www.ncbi.nlm.nih.gov/pmc/articles/PMC2848616> .
```

```
      } .

### Attribution of MP1
:A_MP1= {:A_MP1   a mp:Attribution;
            rdfs:label "A_MP1: MP1 authorship attribution" ;
            mp:atTime "2013-08-01T00:00:00Z" ;
            mp:attributionOfAgent :TClark .
      } .

### Principal Claim
:C1=  {:C1 a mp:Claim ;
            rdfs:label  "C1: Rapamycin inhibits mTOR";
            mp:statement      "Rapamycin [is] an inhibitor of the mTOR
pathway.";
            mp:supportedBy    :Ref5;
            mp:qualifiedBy    obo:CHEBI_68481, obo:INO_0000736;
            mp:hasAttribution :A_C1 .
      } .

### Attribution of C1
:A_C1= {:A_C1     a mp:Attribution;
            rdfs:label "A_C1: C1 authorship attribution" ;
            mp:atTime "2010-01-01T00:00:00Z" ;
            mp:attributionOfAgent :PSpilman .
      } .

### Reference
:Ref5= {:Ref5 a mp:Reference ;
                  rdfs:label "Ref5: Harrison et al. 2009";
                  mp:citation
      "Harrison et al. Nature 2009, 460(7253):392-395." ;
            mp:hasAttribution :A_C1 .
      } .

### End

} .
```

A.2.3 RDF for Example 3, Figure 9

```
@prefix     xsd:  <http://www.w3.org/2001/XMLSchema#> .
@prefix     rdf:  <http://www.w3.org/1999/02/22-rdf-syntax-ns#>.
@prefix     rdfs: <http://www.w3.org/2000/01/rdf-schema#> .
@prefix     rdfg: <http://www.w3.org/2004/03/trix/rdfg-1/>.
@prefix     dct:  <http://purl.org/dc/terms/> .
@prefix     foaf: <http://xmlns.com/foaf/spec/0.98#> .
@prefix     prov: <http://www.w3.org/ns/prov#> .
@prefix     mp:   <http://purl.org/mp/> .
@prefix     :     <http://www.example.com/micropublication/ex3/> .

{

### Agents
:ClarkT     a     mp:Agent, prov:Person, foaf:Person;
            foaf:name   "Tim Clark";
            foaf:mbox_sha1sum
```

```
      "d9c9efcbcb2877d085cff13ec8b748772fec0ed6" .


:SpilmanP   a     mp:Agent, prov:Person, foaf:Person;
            foaf:name   " Patricia Spilman";
            foaf:mbox_sha1sum
      "6b4cacd4bc4de8d5d45903c29dec30eba1d696d3" .

:UofManchester    a     mp:Agent, prov:Organization, foaf:Organization;
            foaf:name
      "University of Manchester, School of Computer Science";
            foaf:homepage     <http://www.cs.manchester.ac.uk/> .

:MGH        a     mp:Agent, prov:Organization, foaf:Organization;
            foaf:name
      "Massachusetts General Hospital";
            foaf:homepage     <http://www.massgeneral.org/> .

### Article Texts
<info:doi/10.1038/nature08221> a mp:ArticleText;
            rdfs:label  "Article Text for Harrison et al. 2009" .

<info:doi/10.1073/pnas.96.6.3228> a mp:ArticleText;
            rdfs:label "Article Text for Hisa et al. 1999".

<http://jaxmice.jax.org/strain/006293.html> a mp:ArticleText;
                        rdfs:label  "Article     Text     for     Mucke's
descriptikon of the PDAPP J20 mouse strain".

<http://dx.doi.org/10.1016/S0165-0173(01)00067-4> a mp:ArticleText;
                        rdfs:label "Article Text for MWM article" .

### Micropublication MP3
:MP3= {:MP3 a mp:Micropublication ;
            rdfs:label
      "MP3: MP Example 3, Digital summary of Spilman et al. 2010";
            mp:asserts        :C3,:A_C3, :S1,:Ref5, :S2, :Ref9, :S3,
                              :Ref10, :D1, :M1, :M2;
            mp:argues         :C3;
            mp:represents
      <http://www.ncbi.nlm.nih.gov/pmc/articles/PMC2848616> ;

###   MP3 authored by
            mp:attributionAsAuthor :A_MP3 ;

###   MP3 published by
            mp:attributionAsPublisher :P_MP3 .
      }.

:A_MP3= {:A_MP1   a mp:Attribution;
            rdfs:label "A_MP3: MP3 authorship attribution" ;
            mp:atTime "2013-08-01T00:00:00Z" ;
            mp:attributionOfAgent :TClark .
      } .

:P_MP3= {:P_MP3   a mp:Attribution;
```

```
            rdfs:label "A_MP3: MP3 publisher attribution" ;
            mp:atTime "2013-08-01T00:00:00Z" ;
            mp:attributionOfAgent :UofManchester, :MGH .
      } .

### Principal Claim
:C3=        {:C3  a                 mp:Claim;
                  rdfs:label        "C3: Inhibition of mTOR…can slow…";
                  rdf:value         "Inhibition of mTOR by rapamycin
can slow or block AD progression in a transgenic mouse model of the
disease.";
                  mp:supportedBy    :S1,:S2,:S3,:D1;
                  mp:attributionAsAuthor :A_C3 .
            } .

:A_C3= {:A_C3     a mp:Attribution;
            rdfs:label "A_C3: C3 authorship attribution" ;
            mp:atTime "2010-01-01T00:00:00Z" ;
            mp:attributionOfAgent :PSpilman .
      } .

:S1=        {:S1  a           mp:Statement;
                  rdfs:label  "S1: Rapamycin inhibits mTOR";
                  mp:statement "Rapamycin [is] an inhibitor of the mTOR
pathway.";
                  mp:supportedBy :Ref5;
                  mp:attributionAsAuthor :A_C3 .
            }.

:REF5=      {:Ref5 a          mp:Reference;
                  rdfs:label  "Ref5: Harrison et al. 2009";
                  mp:citation
                  "Harrison, D.E., et al. (2009) Rapamycin fed late in
      life extends lifespan in genetically heterogeneous mice, Nature,
      460, 392-395." ;
                  mp:supportedBy
                        <info:doi/10.1038/nature08221> ;
                  mp:attributionAsAuthor :A_C3 .
            } .

:S2=        {:S2        a           mp:Statement;
                        rdfs:label  "S2: PDAPP mice accumulate…";
                        mp:statement "PDAPP mice accumulate soluble and
deposited Aβ and develop AD-like synaptic deficits as well as cognitive
impairment and hippocampal atrophy.";
                        mp:supportedBy :Ref9;
                        mp:attributionAsAuthor :A_C3 .
            }.

:Ref9=      {:Ref9      a           mp:Reference;
                        rdfs:label  "Ref9: Hsia et al. 1999";
                        mp:citation "Hsia, A.Y., et al. (1999) Plaque-
independent disruption of neural circuits in Alzheimer’s disease mouse
models, Proceedings of the National Academy of Sciences, 96, 3228-
3233.";
                        mp:supportedBy
```

```
                                <info:doi/10.1073/pnas.96.6.3228> ;
                        mp:attributionAsAuthor :A_C3 .
            }.

:S3=        {:S3        a           mp:Statement;
                        rdfs:label  "S3";
                        mp:statement  "Rapamycin-fed  transgenic  PDAPP
mice showed improved learning (Figure 1a) and memory (Figure 1b). We
observed significant deficits in learning and memory in control-fed
transgenic PDAPP animals.";
                        mp:supportedBy    :Ref10 ;
                        mp:attributionAsAuthor :A_C3 .
            }.

:Ref10=     {:Ref10     a           mp:Reference;
                        rdfs:label "Ref10: Mucke et al. 2000";
                        mp:citation "Mucke, L., et al. (2000) High-
Level Neuronal Expression of Aβ1-42 in Wild-Type Human Amyloid Protein
Precursor Transgenic Mice: Synaptotoxicity without Plaque Formation,
The Journal of Neuroscience, 20, 4050-4058.";
                        mp:supportedBy
                  <http://www.jneurosci.org/content/20/11/4050> ;
                        mp:attributionAsAuthor :A_C3 .
            }.

:D1=  {:D1              a           mp:Data;
                        rdfs:label
      "D1: Graphs from Spilman et al. 2010 Figure 1";
                        mp:value
      <http://www.plosone.org/article/fetchObject.action?uri=info:doi/1
0.1371/journal.pone.0009979.g001&representation=PNG_M>;
                        mp:supportedBy :M1,:M2 ;
                        mp:attributionAsAuthor :A_C3 .
      } .

:M1=        {:M1        a           mp:Procedure;
                        rdfs:label  "M1:  Feeding  and  MWM  testing
procedure for mice in Spilman et al. 2010";
                        mp:value    "We  fed  a  rapamycin-supplemented
diet... or control chow to groups of PDAPP mice and littermate non-
transgenic controls for 13 weeks. At the end of treatment (7 mo),
learning and memory were tested using the Morris water maze." ;
                        mp:supportedBy
                  <http://dx.doi.org/10.1016/S0165-0173(01)00067-4> ;
                        mp:attributionAsAuthor :A_C3  .
            }.

:M2=        {:M2        a           mp:Material;
                        rdfs:label  "M2: PDAPP J20 mice, JAX 006293";
                        mp:supportedBy
                  <http://jaxmice.jax.org/strain/006293.html> ;
                        mp:attributionAsAuthor :A_C3 .
            }.

### End
}.
```

A.2.4 RDF for Example 5, Figure 13

```
@prefix xml:            <http://www.w3.org/XML/1998/namespace> .
@prefix xsd:            <http://www.w3.org/2001/XMLSchema#> .
@prefix rdf:            <http://www.w3.org/1999/02/22-rdf-syntax-ns#> .
@prefix rdfs:           <http://www.w3.org/2000/01/rdf-schema#> .
@prefix dct:            <http://purl.org/dc/terms/> .
@prefix prov:           <http://www.w3.org/ns/prov#> .
@prefix foaf:           <http://xmlns.com/foaf/spec/> .
@prefix mp:             <http://purl.org/mp/> .
@prefix :               <http://www.example.com/micropublication/2> .

{

### Agents
:ClarkT     a     mp:Agent, prov:Person, foaf:Person;
            foaf:name   "Tim Clark";
            foaf:mbox_sha1sum
      "d9c9efcbcb2877d085cff13ec8b748772fec0ed6" .


:SpilmanP   a     mp:Agent, prov:Person, foaf:Person;
            foaf:name   " Patricia Spilman";
            foaf:mbox_sha1sum
      "6b4cacd4bc4de8d5d45903c29dec30eba1d696d3" .

###   micropublication with Attribution :P7 argues Claim :C4_H,

:MP7=       {:MP7       a                 mp:Micropublication;
                        rdfs:label        "MP Example 5";
                        mp:asserts        :C4_H, :A_C4_H ;
                        mp:quotes         :C1,:C1_1,:C4,:C5,:C6 ;
                        mp:argues         :C4_H;
                        mp:represents
            <http://www.ncbi.nlm.nih.gov/pmc/articles/PMC2848616/> ;

###   authored by
                        mp:attributionAsAuthor :A_MP7 .
      }.

:A_MP7= {:A_MP7   a mp:Attribution;
            rdfs:label "A_MP7: MP7 authorship attribution" ;
            mp:atTime "2013-08-01T00:00:00Z" ;
            mp:attributionOfAgent :TClark .
      } .

:C4_H=      {:C4_H      a                 mp:Claim ;
                        rdfs:label        "C4_H: Holotype for
Rapamycin-mTOR interaction ";
                  rdf:value   "Rapamycins inhibit the function of the
mammalian target of rapamycin mTOR.";
                        mp:attributionAsAuthor :A_C4_H ;
                        mp:supportedBy    :C1, :C1_1, :C4, :C5, :C6;
                        mp:holotypeFor    :C1, :C1_1, :C4, :C5, :C6 .
            }.

:A_C4_H= {:A_C4_H       a mp:Attribution;
```

```
            rdfs:label "A_C4_H: C4_H authorship attribution" ;
            mp:atTime "2013-08-01T00:00:00Z" ;
            mp:attributionOfAgent :TClark .
      } .


:C1=        {:C1        a           mp:Claim;
                        rdfs:label  "C1";
            mp:statement "Rapamycin [is] an inhibitor of the mTOR
pathway." .
            }.

:C1_1=      {:C1_1      a           mp:Claim;
                        rdfs:label  "C1_1";
            mp:statement "Rapamycin…an inhibitor of the mTOR pathway…."
.           }.
:C4=        {:C4        a           mp:Claim;
                        rdfs:label  "C4";
            mp:statement "Rapamycins inhibit the function of the
mammalian target of rapamycin (mTOR)…" .
            }.
:C5=        {:C5        a           mp:Claim ;
                        rdfs:label  "C5";
            mp:statement "Rapamycin potently inhibits downstream
signalling from the target of rapamycin (TOR) proteins" .
            }.
:C6         ={:C6       a           mp:Claim ;
                        rdfs:label  "C6";
            mp:statement "RAFT1 is the direct target of FKBP12-
rapamycin" .
            }.
} .
```

REFERENCES

1. Lebo T, Sahoo S, McGuinness D, Belhajjame K, Cheney J, Corsar D, Garijo D, Soiland-Reyes S, Zednik S, Zhao J: **PROV-O: The PROV Ontology W3C Recommendation 30 April 2013**. 2013.
2. Ciccarese P, Soiland-Reyes S, Belhajme K, Gray AJ, Goble C: **PAV ontology: Provenance, Authoring and Versioning**. *Journal of Biomedical Semantics* 2013, **4**(37).
3. Horrocks I, Patel-Schneider PF, Boley H, Tabet S, Grosof B, Dean M: **SWRL: A Semantic Web Rule Language Combining OWL and RuleML: W3C Member Submission 21 May 2004**. 2004.
4. Glimm B, Horridge M, Parsia B, Patel-Schneider PF: **A Syntax for Rules in OWL 2**. In: *OWL Experiences and Directions 2009 (OWLED 2009): 2009; 23-24 October 2009, Chantilly, Virginia, USA*. CEUR Workshop Proceedings.
