# Supplementary material for: Micropublications: a semantic model for claims, evidence, arguments and annotations in biomedical communications
Source: J Biomed Semantics. 2014 Jul 4;5:28. doi: 10.1186/2041-1480-5-28 (PMC4530550; doi:10.1186/2041-1480-5-28)
Supplement: Additional file 3 — Relationship of Micropublications to the SWAN Model. [file 2041-1480-5-28-S3.pdf]

Relationship of Micropublications to the SWAN Model

Additional Material 3 for:
Micropublications: a Semantic Model for Claims, Evidence, Arguments and Annotations in Biomedical Communications

Tim Clark[1,2,3]*, Paolo N. Ciccarese[1,2], Carole A. Goble[3]

[1] Department of Neurology, Massachusetts General Hospital
55 Fruit Street, Boston MA 02114, USA

[2] Harvard Medical School
25 Shattuck Street, Boston, MA 02115 USA

[3] School of Computer Science, University of Manchester
Oxford Road, Manchester M13 9PL, UK

* Corresponding author

Email addresses:
TC: tim_clark@harvard.edu; clarkt@cs.manchester.ac.uk
PNC: paolo.ciccarese@gmail.com
CAG: carole.goble@cs.manchester.ac.uk

A.3 Relationship of Micropublications to the SWAN Model

It may be helpful to outline some of the principal differences and correspondences between the micropublications ontology (mp), and SWAN [1].

- Hypotheses and Micropublications
  - A `swan:Hypothesis` corresponds most closely to an `mp:Micropublication` together with the `Claim` it argues.
- Claims and Statements
  - A `swan:Claim` roughly corresponds to an `mp:Statement`.
- Evidence
  - SWAN models evidence for Claims in the form of literature citations, while Micropublications also model Data and Methods.
  - Micropublications support both literature citations-as-support (document level) as well as direct Claim / Statement citations (statement level).
- Statement Consistency
  - In SWAN, a knowledgebase curator asserts inconsistency, between Claims globally, at a single point.
  - In Micropublications, inconsistency is modeled as *challenge*, and may be asserted by anyone. There may or may not be a global curator, as needed.
- Statement Similarity
  - SWAN implemented the notion of statement similarity using “canonical statements”.

  - Micropublications models statement similarity using similarity groups and uses the holotype of such a group as a surrogate claim to represent the common meaning.
  - Micropublications similarity groups may be asserted as Micropublications themselves.
- Multi-polarity
  - SWAN does not model multipolar argumentation. The concept of inconsistency is vague, relatively subjective, and global.
  - Micropublications models multipolar argumentation, using challenge relationships. It is therefore compatible with bipolar argumentation frameworks. Furthermore, its *challenge* and *support*s relationships are explicitly published with attribution and authority, enabling multiple viewpoints to coexist and be selected/deselected.
- Abstraction and Annotation
  - SWAN and Micropublications both support Comments.
  - Micropublications supports translation of Claims into more or less any useful formal language capable of representing them in the required domain.
  - Micropublications re-formalizations of Claims in other micropublications are also micropublications.
- Layering
  - SWAN does not have formal mechanisms to support layering.
  - Micropublications explicitly support layering. A Micropublications may be constructed about another Micropublications, and this may be layered as deeply as one wishes.
- Grounding
  - The Micropublications model is theoretically grounded in Argumentation Theory. SWAN is not.

REFERENCES

1. Ciccarese P, Wu E, Wong G, Ocana M, Kinoshita J, Ruttenberg A, Clark T: **The SWAN biomedical discourse ontology**. *J Biomed Inform* 2008, **41**(5):739-751.
